# Network Formation via Anion Coordination: Crystal Structures Based on the Interplay of Non-Covalent Interactions

**DOI:** 10.3390/molecules23030572

**Published:** 2018-03-03

**Authors:** Matteo Savastano, Carla Bazzicalupi, Palma Mariani, Antonio Bianchi

**Affiliations:** 1 Department of Chemistry “Ugo Schiff”, via della Lastruccia 3, 50019 Sesto Fiorentino, Italy; matteo.savastano@unifi.it (M.S.); carla.bazzicalupi@unifi.it (C.B.); palma.mariani@unifi.it (P.M.); 2 Consorzio per lo Sviluppo dei Sistemi a Grande Interfase (CSGI), via della Lastruccia 3, 50019 Sesto Fiorentino, Italy

**Keywords:** tetrazines, anion complexes, weak forces, anion–π, halogen–π, crystal structures

## Abstract

We describe the synthesis and the structural characterization of new H_2_L(CF_3_CO_2_)_2_ (**1**) and H_2_L(Ph_2_PO_4_)_2_ (**2**) compounds containing the diprotonated form (H_2_L^2+^) of the tetrazine-based molecule 3,6-di(pyridin-4-yl)-1,2,4,5-tetrazine. X-ray diffraction (XRD) analysis of single crystals of these compounds showed that H_2_L^2+^ displays similar binding properties toward both anions when salt bridge interactions are taken into account. Nevertheless, the different shapes, sizes and functionalities of trifluoroacetate and diphenyl phosphate anions define quite different organization patterns leading to the peculiar crystal lattices of **1** and **2**. These three-dimensional (3D) architectures are self-assembled by a variety of non-covalent forces, among which prominent roles are played by fluorine–π (in **1**) and anion–π (in **2**) interactions.

## 1. Introduction

The bulk macroscopic properties of solid molecular materials are governed by weak non-covalent forces originating from interactions occurring at the molecular and/or atomic level. These weak interactions range greatly in strength and specificity, spanning from the relatively strong hydrogen bonds to the very feeble dispersion forces. Van der Waals, π–π stacking, halogen bond, cation–π and anion–π interactions fall within this range, forming a library of binding forces that can be exploited for materials design and construction [1]. 

**Figure 1:**
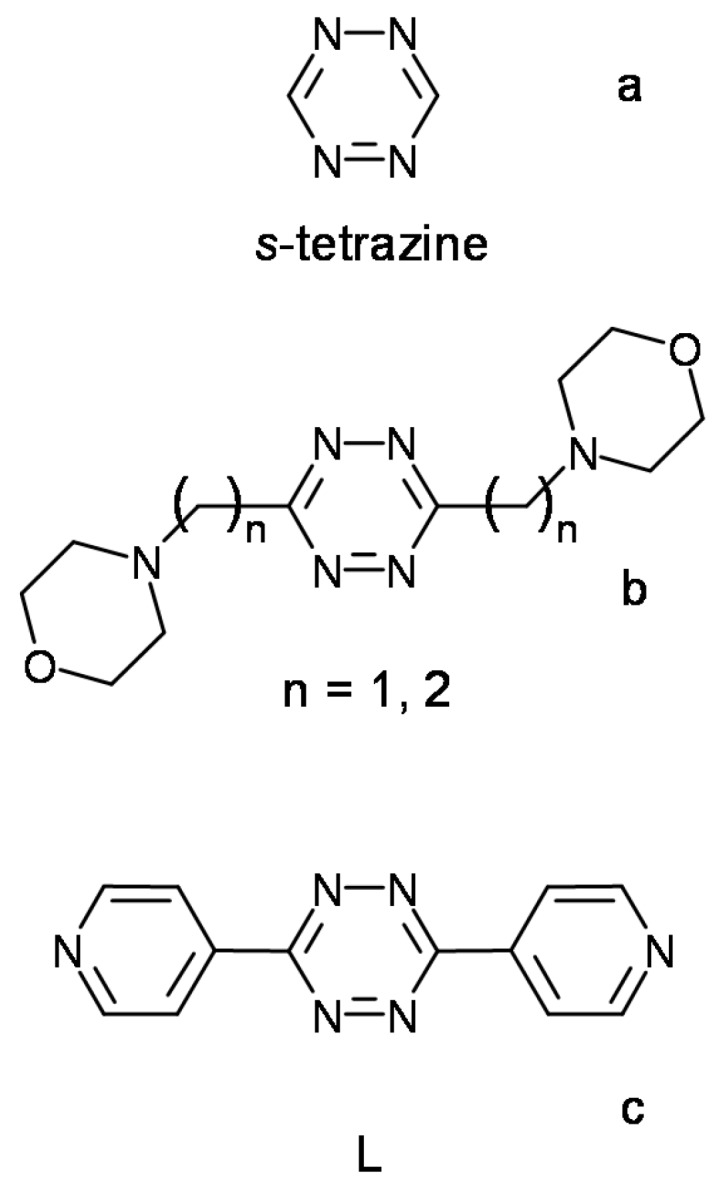
(**a**) *s*-Tetrazine and (**b**) its morpholine and (**c**) pyridine derivatives.

Anion–π interactions [2], that is, anion interactions with aromatic groups, have rapidly become quite popular despite their recent recognition [2,3,4,5,6,7,8,9,10]. Their importance has long been underappreciated as it is counterintuitive to expect that negatively charged species might be attracted by aromatic rings, which are commonly characterized by negative quadrupole moments. Nevertheless, the insertion of strongly electron-withdrawing substituents may invert these quadrupole moments, turning parent aromatic systems into π-acids able to attract anions. A similar effect is brought about by the presence in the aromatic rings of electronegative heteroatoms. This is the case, for instance, of *s*-tetrazine (Figure 1a) which is characterized by a high and positive quadrupole moment (Qzz = 10.7 B). *s*-Tetrazine also displays a high molecular polarizability (α_||_ = 58.7 a.u.), so that an ion-induced polarization term combines with the electrostatic one to enhance its anion–π binding properties [11].

Indeed, in recent reports, we showed that *s*-tetrazines appended with morpholine residues of different lengths (Figure 1b) do bind anions in aqueous solution, forming complexes of appreciable stability with both inorganic [12,13,14,15] and organic [16] anions. Thermodynamic data for the formation of these complexes revealed that their stability is poorly dependent on the charge produced by protonation of the ligand’s morpholine groups and, in several cases, even the neutral (not protonated) ligands are able to bind anions. This peculiar behavior suggested that forces other than charge–charge attractions must be dominant in keeping together the interacting partners [12,13,14,15,16]. Crystal structures of many complexes with inorganic anions evidenced that, in the solid state, these anions are invariably located over the tetrazine ring, at short interaction distances, despite the presence of two ammonium groups in the ligands, that occasionally contribute to bind the anions via salt bridge interactions [12,13,14,15]. Accordingly, it seems that the anion–π interaction is the dominant force in these association processes, even if the flexibility of these ligands opens up the possibility of further contributions (hydrogen bonds, salt bridges) to the stability of their anion complexes, especially in solution. Other non-covalent forces, such as van der Waals (vdW) and π–π stacking interactions, enrich this interplay of weak forces in the case of organic anions bearing aromatic residues [16]. Interestingly, some specific structural aspects inferred for these anion complexes in solution proved to be important constructive elements for the formation of supramolecular networks in the solid state [12,13,14,15,16].

In order to search for different association patterns between anions and tetrazine ligands, we investigated into eliminating the flexibility of the previous molecules (Figure 1b), to force the ammonium groups formed upon protonation to stay as far away as possible from the tetrazine ring, thus preventing the simultaneous intramolecular action of salt bridge and anion–π interactions. To this purpose, we are now considering the rigid and planar tetrazine molecule 3,6-di(pyridin-4-yl)-1,2,4,5-tetrazine (L, Figure 1c), bearing two pyridine residues attached through their 4 position. This molecule, which is known for its use in the construction of metal–organic frameworks (MOFs) and coordination polymers [17,18,19,20,21,22,23,24,25,26,27,28], has never arouse interest as anion receptor, although a few L–anion contacts were discernible in a few crystal structures [24,25,26]. Nevertheless, while the neutral L appears to be an effective element for the construction of MOFs, protonated forms of L combine structural and electronic characteristics that are promising for the formation of interesting supramolecular assemblies with anions.

In order to analyse this issue from a structural point of view, we managed to crystallize H_2_L^2+^ in the presence of two anions of quite different characteristics, in terms of size, shape, and functional groups, like trifluoroacetate (CF_3_CO_2_^−^) and diphenyl phosphate (Ph_2_PO_4_^−^). X-ray diffraction (XRD) analysis of single crystals of H_2_L(CF_3_CO_2_)_2_ and H_2_L(Ph_2_PO_4_)_2_ showed that the protonated form of the ligand H_2_L^2+^ is actually able to form ordered lattices, held together by a variety of weak non-covalent forces, whose overall architectures essentially depend on the anion nature. We report here the results of this study. 

## 2. Results and Discussion

### Crystal Structures of H_2_L(CF_3_CO_2_)_2_ and H_2_L(Ph_2_PO_4_)_2_

The diprotonated form of L, H_2_L^2+^, was crystallized in the presence of trifluoroacetate and diphenylphosphate anions giving rise to H_2_L(CF_3_CO_2_)_2_ and H_2_L(Ph_2_PO_4_)_2_ salts, hereafter indicated as **1** and **2**, respectively. 

**Figure 2:**
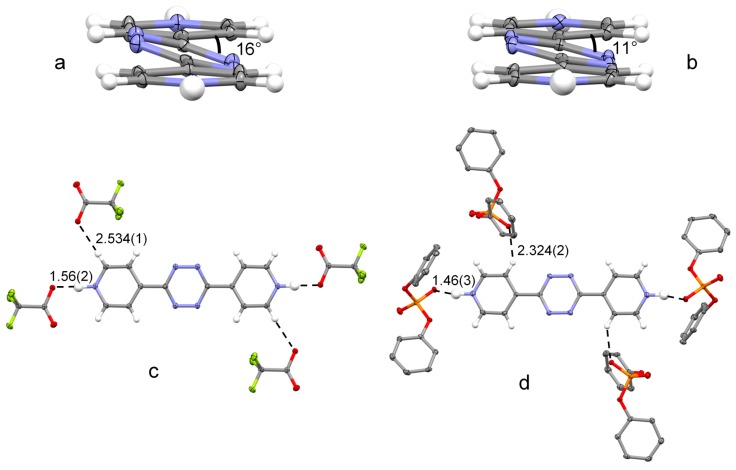
Overall planar conformation assumed by L in (**a**) H_2_L(CF_3_CO_2_)_2_ and (**b**) H_2_L(Ph_2_PO_4_)_2_, and main intermolecular interactions in the crystal packing of (**c**) H_2_L(CF_3_CO_2_)_2_ and (**d**) H_2_L(Ph_2_PO_4_)_2_. All distances are in Å. Thermal ellipsoids are drawn at the 50% probability level. Color code: C, grey; H, white; N, blue; O, red; P, orange; F, greenish yellow.

In both structures, the ligand is centrosymmetric and features all atoms, but the tetrazine nitrogens, almost perfectly on a plane which forms a dihedral angle of 16° and 11°, in **1** and **2** respectively, with the tilted tetrazine ring (Figure 2a,b). As evidenced by crystallographic data, the planar structure with slightly tilted tetrazine ring appears to be the preferred conformation for L. Actually, the dihedral angle (θ) between the two pyridine rings is narrowly distributed close to 0° (median 3.45°, lower and upper quantile 0.005° and 20.091°, respectively), the tetrazine ring being tilted less than 20° in 93% of structures featuring θ smaller than 4° (see Supplementary Materials for Cambridge Structural Database (CSD) search details).

The protonated pyridine groups are involved in the strongest interactions found in the crystal packings, as their NH^+^ groups are involved in very short and linear, or almost linear, NH^+^∙∙∙O^−^ hydrogen bonds (salt bridges) both in **1** (NH∙∙∙O distance, 1.56(2) Å; N–H–O angles, 180(2)°, Figure 2c) and in **2** (NH∙∙∙O distance, 1.46(3) Å; N–H–O angle, 167(3)°, Figure 2d). The C–O∙∙∙H and P–O∙∙∙H angles, respectively 115° and 117°, show that these hydrogen bonds occur in, or very close to the directions of the conventionally viewed oxygen sp^2^ lone pairs. As shown in Figure 2c,d, two hydrogen atoms of the other pair of symmetry related anions are involved in short CH∙∙∙O contacts with two symmetry related aromatic H atoms, of the ligand, in ortho (**1**) or meta positions (**2**) with respect to the protonated pyridine nitrogen. Supplementary Tables S1 and S2 report selected hydrogen bond distances for these structures.

Interestingly, similar strong contacts are present in a crystal structure containing L and trimesic acid, for which the formation of salt bridges is not indicated [29], as well as in the crystal structures of the analogous 2,2′-(1,2,4,5-tetrazine-3,6-diyl)dipyridine protonated ligand in the presence of nitrate [30], perchlorate [31] and tetrabromoterephthalic acid [32].

Thus, the strong salt bridge interactions determine the prime anion binding properties of H_2_L^2+^, which are very similar for both trifluoroacetate and diphenylphosphate (Figure 2c,d), while the specific anion properties (shape, size and functional groups) govern the self-organization pattern of the different elements into the final architectures (packings) of the crystalline compounds **1** and **2**. 

**Figure 3:**
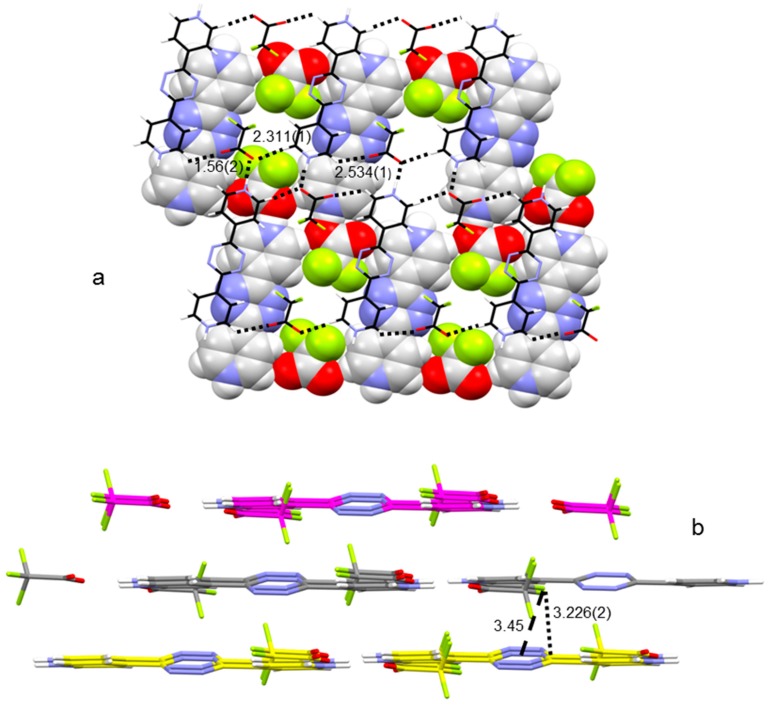
Crystal packing of H_2_L(CF_3_CO_2_)_2_. (**a**) Polymeric diamond-shaped planar grid which develops in the (1−21) crystallographic plane; (**b**) adjacent layers (F∙∙∙ring centroid and shortest F∙∙∙C distances are displayed). All distances are in Å. Color code: (**a**) C, grey; H, white; N, blue; O, red; F, greenish yellow; (**b**) as above, but C is also magenta and yellow, depending on the plane.

Indeed, in the case of H_2_L(CF_3_CO_2_)_2_ (**1**), the C–C bond and one of the C–F bonds of each anion lie on the main ligand plane together with the -CO_2_ group, which joins three distinct ligand molecules (Figure 3a): one oxygen of the carboxylate group bridges two H_2_L^2+^ with a bifurcated NH∙∙∙O∙∙∙HC hydrogen bond, while the other oxygen connects with the pyridine group of a third H_2_L^2+^ via a CH∙∙∙O contact (2.534(1) Å). The inversion symmetry repeats these interactions, giving rise to a polymeric diamond-shaped planar grid which develops in the (1−21) crystallographic plane. Adjacent layers, which are about 3.2 Å apart from each other, are tethered, besides π–π stacking and vdW interactions, by interesting lone pair–π contacts between fluorine atoms of the anion and tetrazine ligand rings (F∙∙∙ring centroid/offset distances, 3.45/1.53 Å; shortest F∙∙∙C contact, 3.226(2) Å; Figure 3b). 

**Figure 4:**
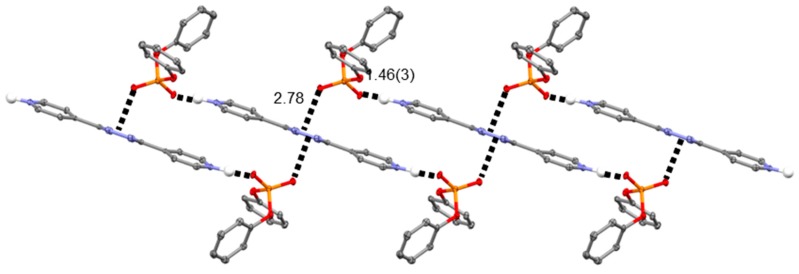
The infinite net ribbon formed by H_2_L^2+^ and diphenylphosphate ions in H_2_L(Ph_2_PO_4_)_2_. N–H∙∙∙O (salt bridge) and O∙∙∙π (anion–π) contacts are displayed. All distances are in Å. Color code: C, grey; H, white; N, blue; O, red; P, orange.

**Figure 5:**
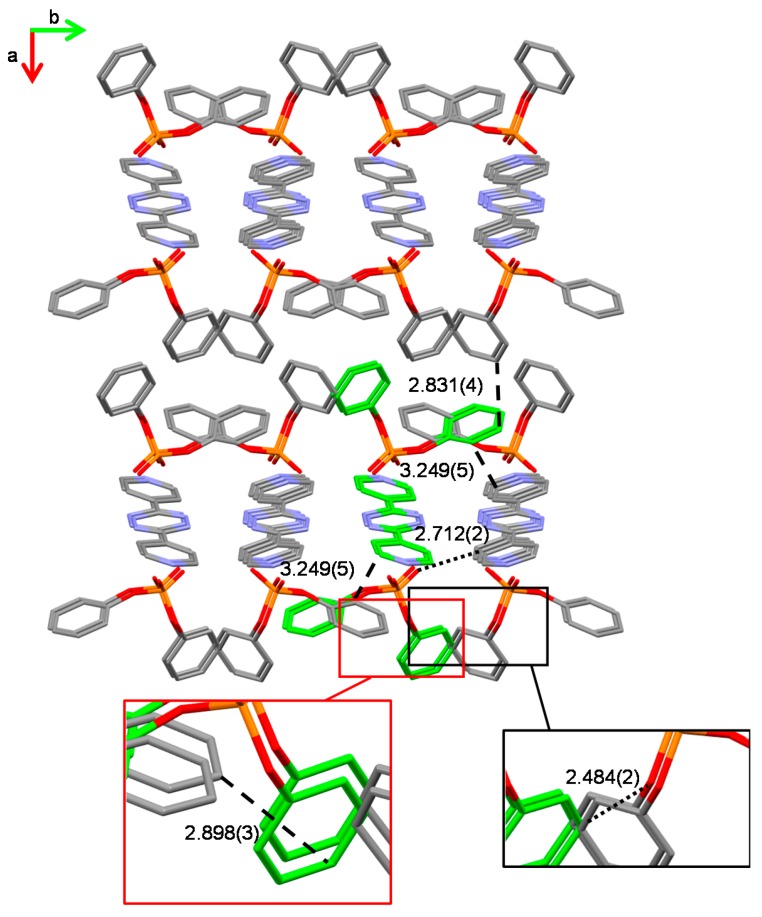
Overall crystal packing in H_2_L(Ph_2_PO_4_)_2_ (hydrogen atoms omitted for clarity). C–H∙∙∙O (dotted lines), CH∙∙∙π and π∙∙∙π contacts (C∙∙∙C distances, dashed lines) are evidenced. All distances are in Å. Green carbon atoms provide a view down the crystallographic *c* axis of a net ribbon like the one in Figure 4. Color code C, grey or green; N, blue; O, red; P, orange.

On the other hand, in the case of H_2_L(Ph_2_PO_4_)_2_ (**2**), the second shorter contact is provided by the anion–π interaction involving a diphenylphosphate, non-esteric oxygen atom and the tetrazine ring (O∙∙∙ring centroid distance/offset, 2.78/0.24 Å). In this structure, each O–P–O group bridges two H_2_L^2+^ protonated molecules, which are replicated by the inversion symmetry in such a way that the N–H∙∙∙O (salt bridge) and O∙∙∙π (anion–π) interactions define rectangular shapes which can be seen as the meshes of an infinite net ribbon (Figure 4). As shown in Figure 5, these ribbons grow along the *c* axis of the crystal packing and are in contact with the adjacent symmetry related ones via CH∙∙∙O interactions involving the above-mentioned ligand carbon atom in meta position with respect to the pyridine nitrogen (2.324(2) Å), as well as a CH group from a close symmetry related anion (2.484(2) Å, inset Figure 5). Moreover, the crystal is further stabilized by a network of CH∙∙∙π and π–π stacking contacts (marked by dashed lines in Figure 5) which mainly involve the phenyl groups of the anions among themselves as well as that of the phenyl groups of the anions and the pyridine groups of the ligand.

## 3. Materials and Methods

### 3.1. General

All starting materials were high purity compounds purchased from commercial sources and were used as supplied. Crystals of H_2_L(CF_3_CO_2_)_2_ and H_2_L(Ph_2_PO_4_)_2_ suitable for XRD analysis were obtained by slow evaporation at room temperature of solutions obtained by treating L (10 mg) in CH_2_Cl_2_ (20 cm^−3^) with 5 eq. of CF_3_CO_2_H and Ph_2_PO_4_H, respectively. Crystals were collected by filtration and air dried. Elem. Anal.: Calc. for C_16_H_10_F_6_N_6_O_4_: C, 41.39; H, 2.17; N, 18.10. Found: C, 41.28; H, 2.12; N, 18.16. Elem. Anal.: Calc. for C_36_H_30_N_6_O_8_P_2_: C, 58.70; H, 4.10; N, 11.41. Found: C, 58.56; H, 4.01; N, 11.50.

### 3.2. Crystal Structure Determination

Crystal data for H_2_L(CF_3_CO_2_)_2_ (**1**), C_16_H_10_F_6_N_6_O_4_ (M = 464.30 g/mol): triclinic, space group *P*1 (No. 2), a = 5.0998(3) Å, b = 9.0745(6) Å, c = 9.9302(6) Å, α = 88.146(5)°, β = 83.905(5)°, γ = 87.252(5)°, V = 456.26(5) Å^3^, Z = 1, T = 298 K, μ(MoKα) = 0.164 mm^−1^, D_calc_ = 1.690 g/cm^3^, 8046 reflections measured (8.6° ≤ 2θ ≤ 58.11°), 2189 unique (R_int_ = 0.0365, R_σ_ = 0.0420) which were used in all calculations. Full-matrix least-square refinements were performed by SHELXL version 2014/7 [33]. The pyridinium hydrogen was localized in the ΔF map, introduced in the calculation and freely refined with isotropic treatment. The final R1 was 0.0429 (I > 2σ(I)) and wR2 was 0.1043 (all data). Cambridge Crystallographic Data Centre(CCDC) 1822663.

Crystal data for H_2_L(Ph_2_PO_4_)_2_ (**2**), C_36_H_30_N_6_O_8_P_2_ (M = 736.60 g/mol): monoclinic, space group *P*2_1_/*c* (No. 14), a = 15.138(2) Å, b = 10.658(1) Å, c = 10.645(1) Å, β = 105.92(1)°, V = 1651.6(3) Å^3^, Z = 2, T = 298 K, μ(MoKα) = 0.197 mm^−1^, D_calc_ = 1.481 g/cm^3^, 12234 reflections measured (2.8° ≤ 2θ ≤ 50.04°), 2912 unique (R_int_ = 0.0786, R_σ_ = 0.1010) which were used in all calculations. Full-matrix least-square refinements were performed by SHELXL Version 2014/7 [33]. The pyridinium hydrogen was localized in the ΔF map, introduced in the calculation and freely refined with isotropic treatment. The final R1 was 0.0536 (I > 2σ(I)) and wR2 was 0.0874 (all data). CCDC 1822664.

## 4. Conclusions

Anionic species are special building blocks for the construction of supramolecular lattices, thanks to their variable structures and to the variety of weak forces that, depending on their components, they can make available in addition to the main attractive interactions produced by their negative charge. The additivity of these non-covalent forces makes the anion–receptor interaction so specific that it assumes the well-defined connotation of anion coordination chemistry in solution. In the solid state, however, such specific anion-receptor interaction can be abandoned in favour of the formation of more extended two-dimensional (2D) and 3D self-assembled networks. 

Indeed, in the crystal structures of H_2_L(CF_3_CO_2_)_2_ (**1**) and H_2_L(Ph_2_PO_4_)_2_ (**2**), the protonated ligand and the anions form all kinds of weak interactions that, in principle, might work together, in a convergent manner, towards the formation of definite anion complexes. Nevertheless, in the crystal lattice of these complexes, these forces organize the construction elements in a divergent manner to build supramolecular extended architectures.

Salt bridges, which are very significant for anion complexes with ammonium ligands in solution [34,35,36,37,38], are pivotal elements also for the assembling of complexes **1** and **2** in the solid state, being the strongest anion–ligand interactions observed in their structures. In **1**, salt bridges are accompanied by hydrogen bonds in the formation of 2D layers, while in **2**, they are reinforced by strong anion–π interactions to form infinite ribbons. Such different behaviours seem mostly to be dictated by the different anion geometries: the planar carboxylate group of the almost flat CF_3_CO_2_^−^ anion fits well into planar 2D arrays, which is hardly achievable by the bulky and tetrahedral Ph_2_PO_4_^−^ anion.

An interplay between non-covalent forces, including π–π stacking and vdW interactions along with unconventional hydrogen bonds and fluoride–π contacts in the case of **1**, organizes these layers and ribbons, respectively, into the 3D networks of the crystalline compounds **1** and **2**.

Indeed, while the more flexible morpholine derivatives of *s*-tetrazine shown in Figure 1 display a marked tendency to form definite anion complexes, the rigid L molecule orientates its anion binding ability towards the formation of extended architectures. Accordingly, in addition to being efficient in the construction of MOFs and coordination polymers, L turns out to be also a promising element for the construction of anion coordination-based frameworks and polymers. 

## Supplementary Materials

The following are available online, CSD search details, Table S1: H_2_L(CF_3_CO_2_)_2_: Non-H atom∙∙∙non-H atom distances (Å) and corresponding distances from hydrogen atoms for contacts discussed in the text, Table S2: H_2_L(Ph_2_PO_4_)_2_: Non-H atom∙∙∙non-H atom distances (Å) and corresponding distances from hydrogen atoms for contacts discussed in the text. CCDC numbers 1822663 and 1822664 contain the supplementary crystallographic data for this paper. These data can be obtained free of charge via http://www.ccdc.cam.ac.uk/conts/retrieving.html (or from the CCDC, 12 Union Road, Cambridge CB2 1EZ, UK; Fax: +44-1223-336033; E-mail: deposit@ccdc.cam.ac.uk).
