# Supplementary material for: Network Formation via Anion Coordination: Crystal Structures Based on the Interplay of Non-Covalent Interactions
Source: Molecules. 2018 Mar 3;23(3):572. doi: 10.3390/molecules23030572 (PMC6017772; doi:10.3390/molecules23030572)
Supplement: Supplementary file 1 [file molecules-23-00572-s001.pdf]

molecules

Network Formation via Anion Coordination: Crystal Structures Based on the Interplay of Non-Covalent Interactions.

Matteo Savastano, Carla Bazzicalupi, Palma Mariani and Antonio Bianchi

Supplementary Materials

CSD search details

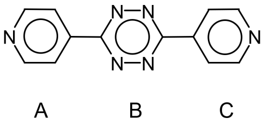

The CSD – system 2017 (version 5.38) has been investigated for ligand L as shown in figure.
The following geometric parameters have been defined:
plane 1: all non-H atoms in pyridine ring A
plane 2: all non-H atoms in pyridine ring C
plane 3: all non-H atoms in pyridine rings A and C
plane 4: all non-H atoms in tetrazine ring B
angle 1: dihedral angle between plane 1 and plane 2
angle 2: dihedral angle between plane 3 and plane 4

Table S1. $H_2L(CF_3CO_2)_2$: non-H atom···non-H atom distances (Å) and corresponding distances from hydrogen atoms for contacts discussed in the text (e.s.d. in parenthesis).

X-H···Y	X···Y distance (Å)	H···Y distance (Å)
N3-H···O1	2.577(2)	1.56(2)
C5-H···O1	3.239(2)	2.311(1)
C4-H···O2	3.155(2)	2.534(1)

Table S2. $H_2L(Ph_2PO_4)_2$: non-H atom···non-H atom distances (Å) and corresponding distances from hydrogen atoms for contacts discussed in the text (e.s.d. in parenthesis).

X-H···Y	X···Y distance (Å)	H···Y distance (Å)
N1-H···O2	2.544(3)	1.46(3)
C4-H···O2	3.113(4)	2.324(2)
C10-H···C16	3.725(4)	2.898(3)
C11-H···C17	3.587(5)	2.831(4)
